# Metabolomic and Lipidomic Analysis of the Colorectal Adenocarcinoma Cell Line HT29 in Hypoxia and Reoxygenation

**DOI:** 10.3390/metabo13070875

**Published:** 2023-07-23

**Authors:** Juan Carlos Alarcon Barrera, Alejandro Ondo-Mendez, Martin Giera, Sarantos Kostidis

**Affiliations:** 1 Center for Proteomics and Metabolomics, Leiden University Medical Center, 2333 ZA Leiden, The Netherlands; j.c.alarcon_barrera@lumc.nl; 2 Escuela de Medicina y Ciencias de la Salud, Universidad del Rosario, Bogotá 111221, Colombia; alejandro.ondo@urosario.edu.co

**Keywords:** hypoxia, reoxygenation, metabolomics, lipidomics, colorectal adenocarcinoma

## Abstract

The poor availability of oxygen and nutrients in malignant tumors drives the activation of various molecular responses and metabolic reprogramming in cancer cells. Hypoxic tumor regions often exhibit resistance to chemotherapy and radiotherapy. One approach to enhance cancer therapy is to indirectly increase tumor oxygen availability through targeted metabolic reprogramming. Thus, understanding the underlying metabolic changes occurring during hypoxia and reoxygenation is crucial for improving therapy efficacy. In this study, we utilized the HT29 colorectal adenocarcinoma cell line as a hypoxia–reoxygenation model to investigate central carbon and lipid metabolism. Through quantitative NMR spectroscopy and flow injection analysis – differential mobility spectroscopy—tandem mass spectrometry (FIA-DMS-MS/MS) analysis, we observed alterations in components of mitochondrial metabolism, redox status, specific lipid classes, and structural characteristics of lipids during hypoxia and up to 24 h of reoxygenation. These findings contribute to our understanding of the metabolic changes occurring during reoxygenation and provide the basis for functional studies aimed at metabolic pathways in cancer cells.

## 1. Introduction

Poor availability of oxygen and blood-circulating nutrients are common characteristics of malignant tumors stemming from an imbalance between angiogenesis and rapid tumor cell growth and proliferation. The local hypoxic and nutrient-deprived microenvironment triggers cancer cells to employ multiple molecular responses like the activation and stabilization of hypoxia-inducible factor (HIF) [1]. These responses induce, or often are caused by, metabolic reprogramming of cancer cells that allows them to access alternative nutrient sources and rewire intracellular metabolic pathways [2,3]. Among the metabolic alterations, the most commonly appearing is increased glycolysis that results in the production of lactate from pyruvate. Albeit less energetically efficient than mitochondrial respiration, increased glycolytic flux characterizes most cancer cells even in conditions of abundant O_2_, a phenomenon known as the Warburg effect [4]. It has been established that stabilization of HIF1α (i.e., the O_2_-dependent α subunit of isoform 1 of HIF) has an important contribution to the Warburg effect by inducing the expression of genes encoding glycolytic enzymes and glucose transporters as well as lactate efflux [5,6]. Besides glycolysis, HIFs also regulate the formation of reactive oxygen species (ROS) in multiple ways, for example by preventing pyruvate contribution to mitochondrial oxidative phosphorylation (OXPHOS) [7,8], and inhibiting de novo lipogenesis. Fatty acids (FAs) are essential for cell survival, contributing to phospholipid biosynthesis, crucial to membrane formation, producing signaling lipids or energy via β-oxidation [9]. Under normoxic conditions, FA biosynthesis proceeds via the production of lipogenic citrate from glucose and glutamine, at the expense of NAD^+^ [10,11]. However, in hypoxic cancer cells, this route of lipid synthesis has a high cost in NAD^+^, the regeneration of which requires the limited O_2_ as the terminal electron acceptor. Thus, tumor cells fulfill their demand for FAs by taking up exogenous lipids and bypass the NAD^+^-consuming pathways [12]. Another factor that contributes to this metabolic switch is the decreased activity of O_2_-dependent desaturases, like stearoyl-CoA desaturase (SCD). Desaturation of FAs by SCD is critical to reduce the cytotoxic effects that accompany the accumulation of saturated FAs, like for example the overproduction of ceramides (CERs) which induce ER stress and apoptosis [9]. As such, hypoxic cancer cells rely on taking up exogenous unsaturated FAs to support the synthesis of complex lipids, which in some tumor types are also stored in lipid droplets in the form of triacylglycerols (TAGs) and cholesterol esters (CEs) [13,14].

Hypoxia-induced metabolic reprogramming and the O_2_-dependent dynamic changes occurring within tumors have emerged as an important area of research to improve the efficacy of cancer therapy [2]. It is well known that hypoxic regions of tumors exhibit resistance to chemotherapy and radiotherapy (RT) [15,16]. Radioresistance in particular occurs via a network of complex mechanisms, including HIF1α signaling, that reduce RT-induced cytotoxic DNA damage [17]. Among the strategies to improve both radiosensitivity and chemosensitivity, one emerging approach is to indirectly increase the availability of O_2_ in the tumor by targeted metabolic reprogramming that aims to lower the utilization of O_2_ in metabolic pathways [18,19]. One prominent example, which is currently being tested in clinical trials of colorectal cancer patients (NCT03053544), is targeting complex I of the electron transport chain (ETC) by metformin [20]. It was found that metformin increases radiosensitivity in two ways, first by increasing toxic ROS production after RT and second by accumulating 2-OG which triggers the degradation of HIF1α and its associated radioresistance-inducing signaling [21]. It is evident from this example that the development of drugs targeting selected metabolic pathways requires knowledge of metabolic changes occurring in cancer cells from normoxia to hypoxia and vice versa during reoxygenation.

Within this framework, in this study, we used the colorectal adenocarcinoma (CRC) cell line HT29 as a model of hypoxia–reoxygenation to study central carbon and lipid metabolism. HT29 cells are highly hypoxic in vivo, and they are characterized by a mutation in the *PIK3CA* gene that encodes the catalytic subunit of phosphatidylinositol 3 kinase-α (PI3Kα) [22]. *PIK3CA* is the most frequently mutated oncogene in human cancer and occurs in more than 30% of colorectal cancers [23,24]. We used quantitative NMR spectroscopy and flow-injection–differential mobility spectroscopy–tandem mass spectrometry (FIA-DMS-MS/MS) to analyze the changes in metabolites and lipids occurring during hypoxia and reoxygenation. We used three distinct time periods for our analysis. There was one short period of 30 m and 1 h, to observe any acute changes in central carbon metabolism, which might occur in parallel and/or due to the rapid degradation of HIF upon oxygen sensing [25,26]. A second (12 h) and a third (24 h) period of extended reoxygenation were aimed at long-term alterations particularly in cellular lipid composition, as short-term reoxygenation up to 1 h exhibited no significant changes in this molecular domain. We show how hypoxic HT29 cells respond to reoxygenation by altering their mitochondrial metabolism, redox status, and remodeling of specific lipid classes.

## 2. Materials and Methods

### 2.1. Cell Cultures

The human colorectal adenocarcinoma cell line HT29 was obtained from ATCC (Manassas, VA, USA). Cell culture media and all additives for cell growth were purchased from Thermo Fisher Scientific (Waltham, MA, USA). A total of 2.5 million cells were seeded per well in six-well plates (Corning, Costar TC-Treated, Merck, Darmstadt, Germany) and cultured at 37 °C with 5% CO_2_ in humidified air, using Dulbecco’s modified Eagle medium (DMEM; 10569010). The cell media were supplemented with penicillin (5000 IU/mL), streptomycin (5 mg/mL) (Pen-Strep, 15140122), and 10% (*v*/*v*) fetal calf serum (FCS, 10270106). To induce hypoxic conditions, the cells were cultured under 1.0% O_2_ in a hypoxia incubator (InVivO_2_-200, Ruskinn Technology, Leeds, UK). Normal growth conditions (control and reoxygenation) were performed at 20% O_2_. Samples of cells representing the hypoxic condition were collected by washing and extraction within 1.5 min of leaving the hypoxia incubator. For the reoxygenation experiment, hypoxic cells were transferred to the normoxic incubator and samples, both cells and conditioned media, were collected at 30 min, 1 h, 12 h, and 24 h. Details regarding the sampling procedures are provided below.

### 2.2. Flow Cytometry Analysis

For flow cytometry analysis, the cells were cultured as described above. After 24 h under hypoxia, the cells were harvested and washed twice with FACS buffer containing PBS and 0.5% FCS. The cells were fixed with fixation buffer (BD Biosciences, Franklin Lakes, NJ, USA) for 20 min at room temperature, followed by a washing step with permeabilization buffer. The cells were then stained with monoclonal phycoerythrin-labeled antibody against HIF-1α (Biolegend, 359704, London, UK). Intracellular expression of HIF-1α was evaluated using flow cytometry (FACSCanto II, BD Biosciences, Franklin Lakes, NJ, USA), and the data analysis was performed using the FlowJo 10.7.1 software.

### 2.3. NMR-Based Metabolomics

NMR-based metabolomics analysis was carried out following a previously published protocol [27]. LC-grade methanol, chloroform, and chemicals for the NMR buffer were purchased from Merck, Darmstadt, Germany, and LC-grade water from Honeywell, Germany. To extract extracellular metabolites, 0.2 mL of conditioned culture medium was collected from each well and immediately mixed with 0.4 mL of pre-chilled (−80 °C) methanol. The mixture was vortexed for 10 s and placed on dry ice for at least 30 min to allow for protein precipitation. Extracellular metabolites were obtained by centrifuging the mixture at 16,000× *g* at −4 °C for 20 min. To extract intracellular metabolites, the culture media were removed from each well, and the cells were washed once with phosphate-buffered saline (PBS) at 37 °C. The cells were then snap frozen with liquid nitrogen to arrest metabolism. To extract polar metabolites, the cells were mixed with a pre-chilled (−20 °C) solution of methanol/chloroform/water (6.75:0.75:2.5, *v*/*v*/*v*) and centrifuged at 16,000× *g* at −4 °C for 10 min. The remaining cell pellet was stored for protein quantification. The supernatants of both extracellular and intracellular metabolites were dried using a gentle nitrogen stream and stored at −80 °C until further analysis.

For NMR measurements, the dried extracts were dissolved in 250 µL of 0.15 M K_2_HPO_4_/KH_2_PO_4_ buffer (pH 7.4) in 99.9% deuterated water (^2^H_2_O), which included 0.2 mM NaN_3_ and 0.05 mM or 0.2 mM trimethylsilylpropionic-*d*_4_ acid sodium salt (TSP; Cambridge Isotope Laboratories, Tewksbury, MA, USA) for cell extracts or culture medium extracts, respectively. Samples were transferred to 3 mm NMR tubes (Bruker Corporation, Billerica, MA, USA) and measured in a 14.1 T Avance Neo NMR spectrometer (Bruker). A 1D ^1^H spectrum was collected per sample using the *noesygppr1d* pulse sequence (TopSpin 3.0) with a mixing time of 0.01 ms, a relaxation delay of 4 s, an acquisition time of 2.65 s, and a spectral width of 12 kHz (20 ppm for ^1^H). The spectral data were processed and referenced to TSP methyl protons at 0.00 ppm and subsequently imported into Chenomx NMR Suite 8 (Chenomx, Edmonton, AB, Canada) for metabolite quantification. Metabolite assignment was based on the Chenomx database as well as 2D NMR experiments (TOCSY and HSQC) of pooled samples. All NMR concentrations (μM or nmoles) were corrected based on the protein mass of each sample. For extracellular metabolites, the quantities of metabolites excreted or consumed by the cells were calculated by subtracting the quantities from blank cell-free cultures under the same conditions. Positive values of nmoles/μg of protein indicate excretion (efflux), while negative values indicate consumption (influx).

### 2.4. FIA-DMS-MS/MS-Based Lipidomics

Comprehensive quantitative lipidomics analysis was conducted using the Shotgun Lipidomics Assistant (SLA) [28]. All chemicals and consumables were of LC-MS grade or higher. Methanol and ammonium acetate were purchased from Merck, Darmstadt, Germany. Dichloromethane (DCM), methyl-*tert*-butyl ether (MTBE), 1-Propanol, and LC-MS-grade water were purchased from Honeywell, Seelze, Germany. Lipids were extracted using liquid–liquid extraction as previously described [29]. Briefly, conditioned media were removed, and cells were washed with PBS. Then, 0.16 mL of methanol was added to each well, and cells were scraped and transferred to microcentrifuge tubes. Lipids were extracted by adding 0.5 mL of methyl tert-butyl ether (MTBE), 0.2 mL of water, and 0.1 mL of internal standard mix (IS; Lipidyzer™ internal standard kit, containing > 50 labeled internal standards for 13 lipid classes; Sciex, Framingham, MA, USA). Samples were vortexed for 30 min at room temperature and centrifuged for 5 min at 16,000× *g* at 20 °C. The upper organic layer was collected and transferred to a glass vial. The extraction was repeated on the remaining pellet with 0.1 mL of methanol, 0.3 mL of MTBE, and 0.1 mL of water. The organic layers from the two extractions were combined, dried under a gentle stream of nitrogen, and pellets stored for protein quantification. Samples for FIA-DMS-MS/MS were resuspended with 0.25 mL of 10 mM ammonium acetate in 1:1 (*v*/*v*) methanol/dichloromethane.

Acquisition and quantification of lipids were performed using the SLA platform on a QTrap 5500 mass spectrometer (Sciex, Framingham, MA, USA) with differential mobility spectrometry (DMS) interface, coupled to a Shimadzu Nexera X2 LC system. The quantitation of lipids was performed as described previously [30,31]. In total, 13 lipid classes, including 850 lipid species, were quantified. Following quality control analysis using pooled samples, the data for 513 lipid species were kept for further analysis. The lipid classes included cholesterol esters (CEs), ceramides (CERs), diacylglycerols (DAGs), dihydroxyceramides (DCERs), free fatty acids (FFAs), hexosylceramides (HCERs), lactosylceramides (LCERs), lysophosphatidylcholines (LPCs), lysophosphatidylethanolamines (LPEs), phosphatidylcholines (PCs), phosphatidyl-ethanolamines (PEs), sphingomyelins (SMs), and triacylglycerols (TAGs). For ease of data analysis, we kept the original lipid nomenclature of the SLA platform. A translation to the current Lipidmaps shorthand notation can be found in [32].

### 2.5. Protein Quantitation

The protein pellets obtained after metabolite and lipid extraction were dissolved in 500 µL of SDS 2% (*w*/*v*) solution and sonicated for 1 h at 60 °C. The samples were then vortexed and centrifugated for 5 min at 16,000× *g* at −4 °C. Protein was determined using the Pierce™ BCA Protein Assay Kit (Thermo Fisher Scientific, Waltham, MA, USA) following the protocol of the manufacturer.

### 2.6. Data Analysis

Data analysis was performed in GraphPad Prism Version 9.5.1. Statistical analysis of all datasets included one-way ANOVA using Brown–Forsythe and Welch ANOVA tests. Follow-up multiple comparisons of pairs were performed using the Dunnett test, with a confidence interval of 95% for adjustment of calculated *p*-values. All reported *p*-values and statistical significance represent adjusted *p*-values unless otherwise stated. Statistical significance without multiple comparisons (*p*-values without multiplicity adjustment) was calculated by the unpaired Welch *t*-test. Heatmaps, Venn diagrams, and volcano plots of metabolites and lipids were generated in R (R Core Team (2023). R: A Language and Environment for Statistical Computing. R Foundation for Statistical Computing. Vienna, Austria. https://www.R-project.org/, (accessed on 10 April 2023) using the R packages *pheatmap*, *RColorBrewer*, *factoextra*, *ggplot2*, and *ggVennDiagram*. The saturation index (SI) was calculated from the total number of saturated acyl chains divided by the total number of unsaturated. For example, in DAGs which have two acyl chains, the total saturated number was the sum of DAGs with saturated FAs over the sum of DAGs with unsaturated FAs. For each DAG used in the equation, if both acyl chains were saturated, then its concentration was multiplied by 2. If one acyl chain was saturated, the concentration was multiplied by 1. If none was saturated, then the concentration is multiplied by 0. Similarly, for the sum of unsaturated DAG in the denominator, the concentration of each DAG was multiplied by 0 if all FAs were saturated, by 1 if one of two is unsaturated, and by 2 if both acyl chains are unsaturated.

## 3. Results

### 3.1. Influence of Hypoxia on Metabolism Compared to Normal Growth Conditions

**Figure 1:**
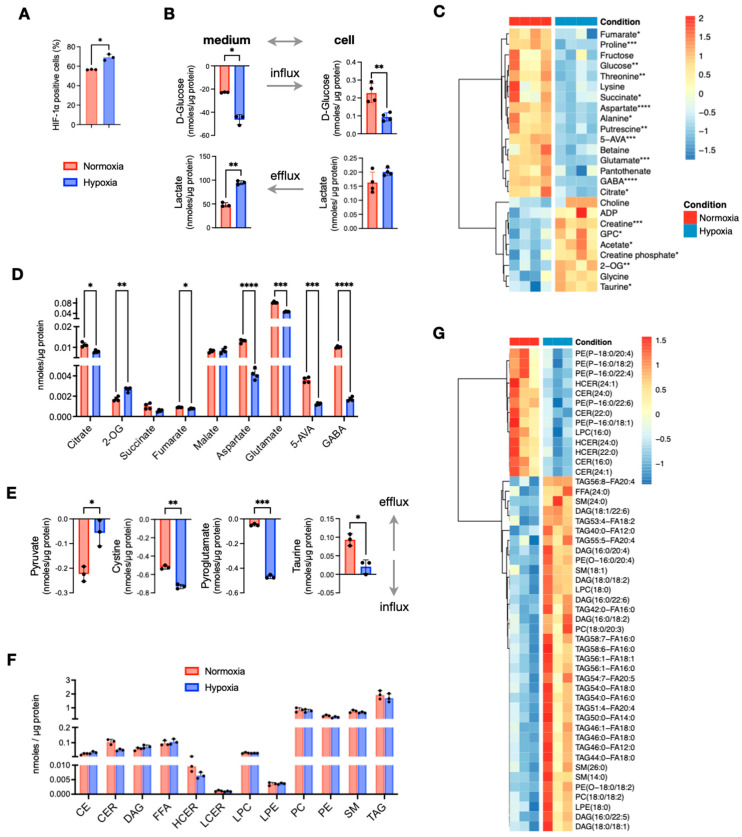
Comparison of in vitro cultured HT29 cells in normoxia and hypoxia. (**A**) Expression of HIF1α measured the number of cell positive (%) to HIF1α selective antibody. (**B**) Quantities of extracellular and intracellular D-glucose and lactate. (**C**) Heatmap of metabolites with *p* < 0.05 (before multiple comparison of pairs correction); the scale represents standardized metabolite concentrations. (**D**) TCA and linked to TCA metabolites. (**E**) Influx and efflux differences of extracellular metabolites. (**F**) Total lipid classes concentrations. (**G**) Heatmap of standardized concentrations of significant lipid species (*p* < 0.05 before multiple testing correction). Star annotations indicate statistical significance after multiple comparison of pairs correction, * *p* < 0.05, ** *p* < 0.01, *** *p* < 0.001, **** *p* < 0.0001.

To investigate the effects of O_2_ deprivation on central carbon and lipid metabolism, we cultured the CRC cell line HT29 under hypoxic (1% O_2_) and normoxic (20% O_2_) conditions. As expected, hypoxic cells showed a significant increase in HIF1α expression (Figure 1A). Using NMR spectroscopy, we quantified the concentrations of polar metabolites and found that hypoxic HT29 cells were more glycolytic, as indicated by the increased consumption of glucose and secretion of lactate compared to normoxic cells (Figure 1B). Several other metabolites of central carbon metabolism also differed significantly (Figure 1C). Among them, the most prominent differences were found for 4-aminobutyrate (GABA), 5-aminovalerate (5-AVA), aspartate, glutamate, proline, putrescine, and threonine that were significantly lower in hypoxic cells, while 2-oxoglutarate (2-OG) creatine and taurine were increased (Figure 1C,D). Interestingly, 2-OG was the only tricarboxylic acid (TCA) cycle intermediate to be increased in hypoxic cells, as all other carboxylic acids were decreased (Figure 1D). From the analysis of the conditioned culture media, we found overall small differences in the consumption of extracellular nutrients between normoxic and hypoxic HT29. A notable exception was pyruvate, which was less consumed from the hypoxic cells, although it is a nutrient that could be used to fuel TCA cycle (Figure 1E). On the other hand, hypoxic cells take up significantly more cystine and pyroglutamate (Figure 1E). Both nutrients can be used for the synthesis of glutathione for which we did not detect a significant difference, while cystine is also a potential source for the synthesis of taurine. The latter had a higher efflux in normoxia (Figure 1E) but had significantly higher intracellular concentrations in hypoxia (Figure 1C).

To assess how the lack of oxygen affected lipid metabolism, we used quantitative targeted lipid analysis by FIA-DMS-MS/MS. Out of 850 quantified lipid species, 513 from 13 lipid classes passed quality control and were further analyzed. We first compared the total concentrations of the lipid classes and observed a noticeable decrease in total ceramides (CERs) and hexosylceramides (HCERs), while differences in all other lipid classes were negligible (Figure 1F). Next, we examined the concentrations of all 513 lipid species, and observed a small subset of 49 lipids to have significant differences (*p* < 0.05) (Figure 1G). Among them, all detected CERs and HCERs were the most decreased lipids in hypoxia. In addition to CERs and HCERs, we found that five phosphatidyl-P-ethanolamines (PE(P)s) were decreased by the lack of O_2_. On the other hand, several diacylglycerols (DAGs), a small number of triacylglycerols (TAGs), two phosphatidylcholines (PCs), two phosphatidyl-O-ethanolamines (PE(O)s), and four sphingomyelins (SMs) were significantly increased under hypoxia. It should be noted that none of these differences remained statistically significant after multiple testing correction. Taken together, out of a large panel of lipids, just about 10% of them exhibited noticeable differences; thus, we conclude that hypoxia was not an important factor for the total lipid content of HT29 cells.

In summary, growing cells under O_2_ deprivation induces metabolic re-wiring that occurs mainly in metabolites of the central carbon metabolism like increased glycolysis and decreased TCA cycle intermediates except 2-OG, whereas lipid metabolism was less affected.

### 3.2. Acute Reoxygenation Is Not Sufficient to Cancel or Reverse the Hypoxia-Induced Effects on Metabolism

**Figure 2:**
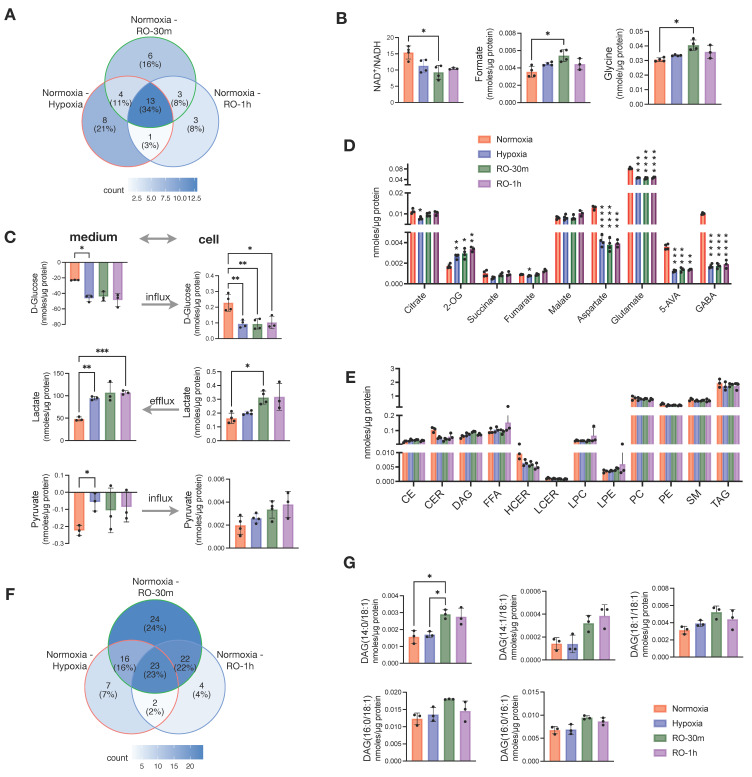
Effects of short reoxygenation up to 1h (RO-30m and RO-1h) in metabolites and lipids of hypoxic HT29 cells and comparison with normoxic HT29. (**A**) Distribution of metabolites with *p* < 0.05 (before multiple comparison of pairs correction) in pairwise comparisons of normoxia–hypoxia, normoxia–RO-30m, and normoxia–RO-1h. (**B**) Significant enhancement of differences occurred between normoxia and hypoxia after short reoxygenation. (**C**) Influx and efflux of D-glucose and lactate, respectively, and their corresponding intracellular concentrations. (**D**) Quantities of TCA intermediates and metabolites linked to TCA; statistical significance is indicated for comparisons against normoxia. (**E**) Quantities of total lipid classes; statistical significance is indicated for comparisons against normoxia. (**F**) Distribution of metabolites with *p* < 0.05 (before multiple testing correction) in pairwise comparisons of normoxia–hypoxia, normoxia–RO-30m, and normoxia–RO-1h. (**G**) DAG species for which reoxygenation enhanced the differences seen between normoxia and hypoxia. Star annotations indicate statistical significance after multiple comparison of pairs correction, * *p* < 0.05, ** *p* < 0.01, *** *p* < 0.001, **** *p* < 0.0001.

Next, we aimed to evaluate whether a short supply of oxygen—for up to 1 h—could have any effect on the metabolism of the cells grown under hypoxia. We applied the same analytical methods and collected data from reoxygenated cells at 30 min (RO-30m) and 1 h (RO-1h) post-hypoxia. The data were then compared to the parallel culture of normoxic cells. Overall, we found that most of the differences we reported above between hypoxia and normoxia were not affected by oxygen supply up to 1 h (Figure S1). When looking at all the pairwise comparisons between normoxia and hypoxia, normoxia and RO-30m, and normoxia and RO-1h, 38 metabolites exhibited a significant change (*p* < 0.05; no multiple testing correction) (Figure 2A). Among them, 26 were in the pair of normoxia–hypoxia, and just 12 metabolites were unique for the two pairs comparing normoxia to either RO-30m or RO-1h (Figure 2A and Figure S1). In addition, out of these 12 metabolites, only glycine and formate remained statistically significant after multiple testing correction (Figure 2B). Among the differences that occurred in reoxygenation, we observed an increase in NADH, which in turn induced a significant change in the ratio of NAD^+^/NADH (Figure 2B) and thus to the redox status of HT29.

Besides the few new changes, reoxygenated cells continued to consume more extracellular glucose and excrete significantly more lactate (Figure 2C). However, we also observed a buildup of intracellular lactate after 30 min of reoxygenation, as well as an increase in intracellular pyruvate without an obvious change in its uptake from the culture medium. (Figure 2C). In addition, GABA, 5-AVA aspartate, and glutamate were still significantly lower in both RO-30m and RO-1h compared to normoxia (Figure 2D). Although these four metabolites are linked to the TCA cycle via oxaloacetate and 2-OG, we found that the other TCA cycle intermediates (fumarate, malate, succinate, citrate, and 2-OG) were increased in reoxygenated cells (Figure 2D). This finding is well in line with the increase in intracellular pyruvate and points to a possible increase in TCA cycle activity due to the restoration of O_2_ supply.

Analysis of the lipidome from the same samples revealed that 1h of reoxygenation did not alter any of the changes observed between hypoxic and control cells in the total concentration of lipid classes (Figure 2E). Although we found half (50%) of the significant pairwise comparisons between normoxia and hypoxia, normoxia and RO-30m, and normoxia and RO-1h to be due to reoxygenation (Figure 2F), careful examination revealed that most of them are due to further increase in TAGs that were already higher in hypoxia compared to normoxia (Figure S2). An exception was five DAG species which had identical concentrations in hypoxia and normoxia but significantly increased in RO-30m (Figure 2G). Interestingly, four of them contained the 18:1 alkyl chain and one the 16:1, possibly pinpointing to a restored activity of SCD1.

Overall, our data showed that a short period of reoxygenation up to one hour induced subtle changes in central carbon metabolism, as for example increased mitochondrial activity and increased availability of intracellular lactate and pyruvate, but did not have any prominent effect on the cells lipidome.

### 3.3. Reoxygenation for 24 h Post-Hypoxia Induces a Metabolic Shift

**Figure 3:**
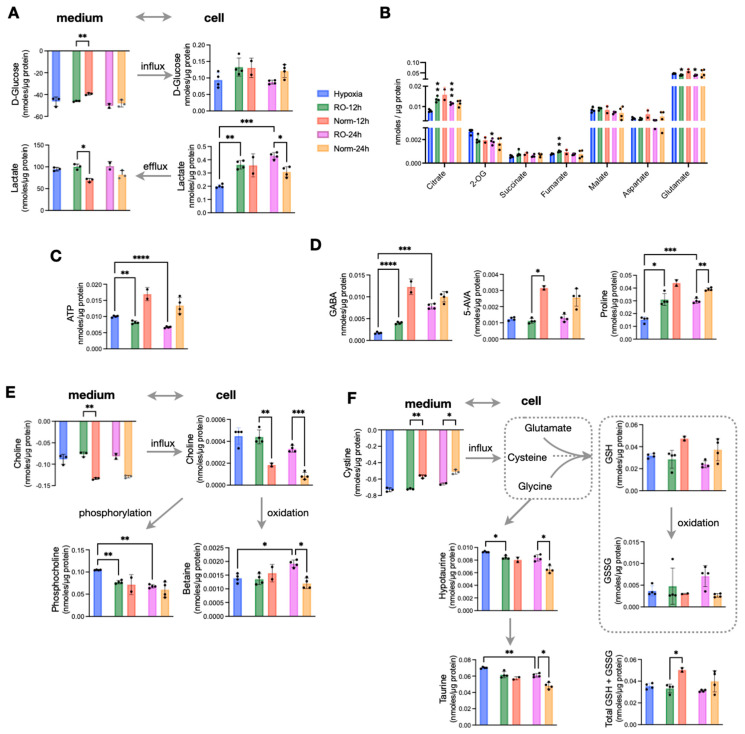
Effects of prolonged reoxygenation up to 24 h in metabolism of hypoxic HT29 cells (RO-12h and RO-24h) and comparisons with parallel normoxic culture of HT29 for the same time period (Norm-12h and Norm-24h). (**A**) Influx and efflux of D-glucose and lactate, respectively, and their corresponding intracellular concentrations. (**B**) Quantities of TCA intermediates and metabolites linked to TCA; statistical significance is indicated for comparisons against hypoxia. (**C**) Intracellular ATP and (**D**) intracellular pools of GABA, 5-AVA, and Proline. (**E**) Influx of choline, its intracellular levels, and the products of its oxidation (betaine) and phosphorylation (phosphocholine). (**F**) Influx of cystine to fuel intracellular cysteine, levels of reduced and oxidized glutathione as well as their sum, and taurine metabolism. Star annotations indicate statistical significance after multiple comparison of pairs correction, * *p* < 0.05, ** *p* < 0.01, *** *p* < 0.001, **** *p* < 0.0001.

An extended O_2_ supply following hypoxia would give sufficient time for hypoxic cells to adapt to a normoxic environment. Thus, we set out to measure whether prolonged reoxygenation of HT29 cells result in adopting the metabolic characteristics of normoxic cells or retain a hypoxia-induced phenotype. To address this, we analyzed samples at 12 h and 24 h of reoxygenation. We then compared them to the data obtained from hypoxic cells as well as with a parallel normoxic culture. We found that reoxygenated cells maintained a higher lactate efflux both at 12 h and 24 h, while surprisingly, they also accumulated significantly more intracellular lactate compared to both hypoxic and normoxic cells at all studied time points (Figure 3A). On the other hand, all TCA cycle intermediates were comparable between normoxic and reoxygenated cells. Those TCA metabolites with lower concentrations in hypoxia accumulated to reach normoxic levels, while 2-OG that was higher in hypoxia was reduced to normoxic levels (Figure 3B). Aspartate and glutamate that had significantly lower concentrations in hypoxia were further reduced during reoxygenation and remained lower compared to both Norm-12h and Norm-24h (Figure 3B). Based on the increase in TCA cycle intermediates and (intracellular and extracellular) lactate build up upon O_2_ restoration, we would also expect an increase in ATP production. Strikingly, we found that ATP was significantly lower in reoxygenated cells compared to Norm-12h and Norm-24h (Figure 3C). In addition, creatine phosphate which can alternatively provide high-energy phosphates was also reduced compared to hypoxia, despite the accumulation of its precursor, creatine in RO-12h and RO-24h (Figure S3).

We then looked at some of the most prominent differences we found in hypoxic cells, and we observed that GABA (Figure 3D) and alanine (Figure S3) were increased to reach normoxic levels after 24 h of O_2_ restoration. However, this was not the case for the other two most hypoxia-affected metabolites, 5-AVA and proline, which remained significantly lower in reoxygenated cells compared to Norm-12h and Norm-24h (Figure 3D). Interestingly, reoxygenation induced a shift in choline metabolism, with RO-12h and RO-24h taking up significantly less choline from the medium but also having much higher intracellular pools. We found that intracellular choline was further oxidized to betaine, especially at 24 h at the expense of reduced phosphorylation to form phosphocholine (Figure 3E). More to that, reoxygenated cells also consumed more cystine, the precursor of intracellular cysteine, which is a basic unit to synthesize glutathione. Similar to choline oxidation, we found increased oxidized glutathione (GSSG) compared to reduced glutathione (GSH) (Figure 3F). However, when we measured the total glutathione (GSH + GSSG), we found that the significant difference at 12 h was attenuated at 24 h, with reoxygenated cells having similar concentrations to normoxic cells (Figure 3F). Finally, both hypotaurine and its product taurine were increased in RO-12h and RO-24h. As these metabolites can be formed from intracellular cysteine, this might explain the increased cystine uptake from reoxygenated cells to support both taurine (mostly) and glutathione (secondary) synthesis.

Taking all these observations together, we conclude that prolonged reoxygenation altered the energetic state of the cells and favored oxidation in the TCA cycle and in choline metabolism. Finally, the increase of GSSG and taurine point to a possible reoxygenation-induced increase of ROS.

### 3.4. Hypoxia–Reoxygenation Affects the Saturation Rather Than the Total Lipid Pool

**Figure 4:**
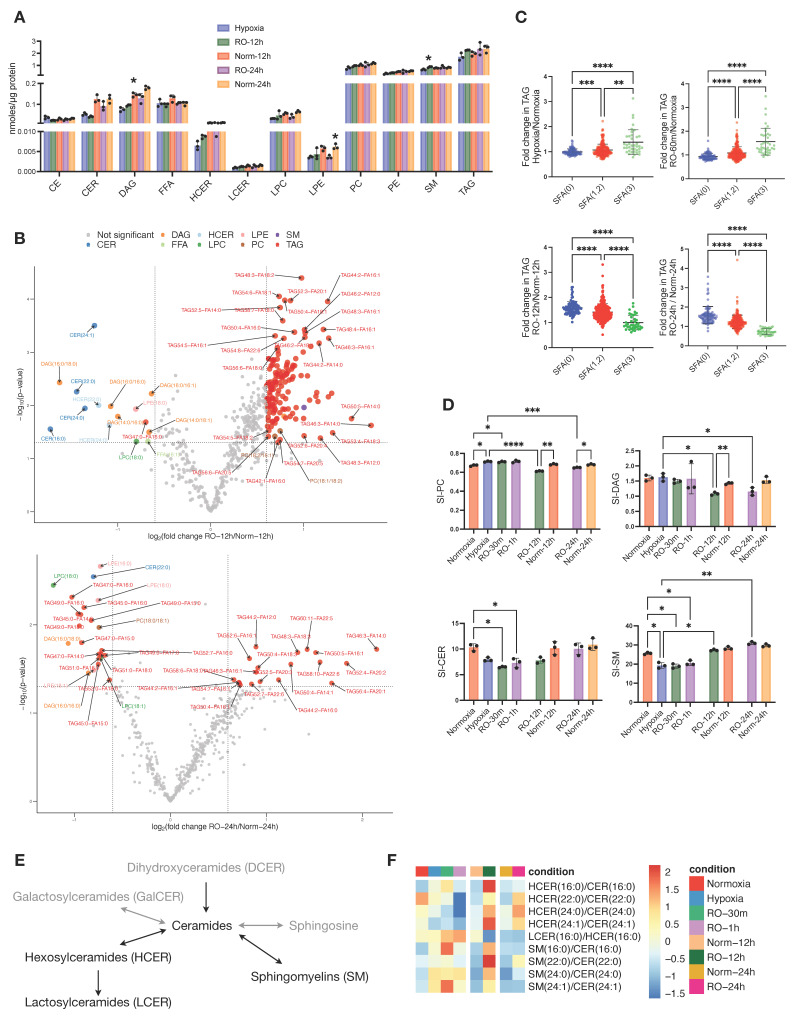
Effects of prolonged reoxygenation up to 24 h in lipid metabolism of hypoxic HT29 cells (RO-12h and RO-24h) and comparisons with parallel normoxic culture of HT29 (Norm-12h and Norm-24h). (**A**) Quantities of total lipid classes; stars in bars of RO-12h and RO-24h indicate statistical significance against hypoxia, whereas stars in bars of Norm-12h and Norm-24h indicate statistical significance against RO-12h and RO-24h, respectively. (**B**) Volcano plots of lipid species for pairwise comparisons of RO-12h–Norm-12h and RO-24h–Norm-24h. (**C**) Fold change of total alkyl chain saturation in TAG for the pairwise comparisons between Hypoxia–Normoxia, RO-1h–Normoxia, RO-12h–Norm-12h, and RO-24h–Norm-24h. SFA(0) represents TAGs with no saturated alkyl chains; SFA(1,2), TAGs with 1 or 2 saturated alkyl chains; and SFA(3), TAGs with all alkyl chains being saturated. (**D**) Saturation index (SI) for DAG, PC, CE, SM, and CER; for SI definition, see Methods. (**E**) Schematic representation of sphingolipid (SL) metabolism; black and grey color correspond to lipid classes measured or not measured in this study, respectively. (**F**) Heatmap of standardized product/substrate ratios of quantified SL species. Star annotations indicate statistical significance after multiple comparison of pairs correction, * *p* < 0.05, ** *p* < 0.01, *** *p* < 0.001, **** *p* < 0.0001.

Following the differences we found in the metabolic state of hypoxic cells after 24 h reoxygenation, we investigated whether the lipidome was altered too. The total concentrations of CER, HCER, DAG, and LPE were lower in RO-12h and RO-24h compared to Norm-12h and Norm-24h, respectively, and except for HCER and DAG, they were also comparable with hypoxic cells. We did not find any noticeable differences for the remaining lipid classes (Figure 4A). When we investigated the concentrations of the lipid species specifically, we found several DAGs, CERs, LPCs, and LPEs significantly decreased in RO-12h, whereas a subset of TAGs was increased (Figure 4B, top). After 24 h of reoxygenation, we observed fewer significant differences between RO-24h and Norm-24h than at 12 h. At this point, a smaller subset of TAGs was increased in RO-24h, while another small subset of TAGs together with two LPCs, 3LPEs, one PC, two DAGs, and one PC exhibited a greater decrease in RO-24h (Figure 4B, bottom). Interestingly, after careful examination of these differences, we observed that most of the decreased lipids in reoxygenated cells were carrying saturated acyl chains, whereas most of the increased lipids were carrying unsaturated or polyunsaturated acyl chains (Figure 4B bottom). Thus, we set out to investigate whether this discrimination was persistent throughout the whole experiment or whether it was due to an alteration in the lipidome, specific to hypoxia or reoxygenation. We calculated the saturation level for all TAGs, i.e., the double bonds present on the three alkyl chains of TAG, and we found that hypoxic cells had significantly higher saturated TAG compared to normoxic cells (Figure 4C). The same was true for reoxygenated cells up to 1h. However, as O_2_ restoration occurred for longer time, TAG in RO-12h and RO-24h became less saturated (*p* < 0.0001) compared to the Norm-12h and Norm-24h, respectively. Considering that the total concentration of TAG in all time points (hypoxia, RO-30m, RO-1h, RO-12h, and RO-24h) was comparable to the concentration in the corresponding normoxic cells (Figure 1F, Figure 2E, and Figure 4A), we can conclude that hypoxia and reoxygenation mainly affect the saturation level of TAG rather than the total intracellular pool of TAG.

Based on this observation, we next calculated the saturation index (SI) of all other lipid classes (Figure 4D and Figure S4). Similarly to TAG, we found PC to be more saturated during hypoxia and short reoxygenation but significantly less saturated after long O_2_ restoration (Figure 4D). DAG and CE also exhibited a lower SI at RO-12h and RO-24h, but there was no difference during hypoxia and at RO-30m and RO-1h. Finally, we found that SM and CER, in contrast to DAG and TAG, were significantly less saturated in hypoxia and short reoxygenation, but these SI differences were attenuated after long O_2_ restoration. 

CERs constitute a critical hub in sphingolipid (SL) metabolism [33] (Figure 4E), and we have seen here that the CER class was more affected by hypoxia than other lipid classes (Figure 1F). Thus, we set out to further investigate CER metabolism by first analyzing the substrate/product ratios between CER, HCER, LCER, and SM lipid species (Figure 4F). We observed that all ratios of HCER/CER, except 16:0, were lower in hypoxia and reoxygenation up to 1h compared to normoxia, but at 12 h and 24 h, these ratios were higher in reoxygenated cells. LCER(16:0) production from HCER(16:0), as well as all SM/CER ratios, were increased in hypoxic cells and up to 1h of reoxygenation and then exhibited a similar ratio to normoxic cells at 12 h (LCER/HCER) and 24 h (both LCER/HCER and SM/CER). We next investigated the composition of each SL class, as expressed by the ratio of each lipid species to the total class (Figure S5). Apart from CER (16:0), the remaining CER with acyl chains of 22:0, 24:0, and 24:1 had higher ratios in hypoxia and up to 12 h of reoxygenation and then became similar to normoxic cells at 24 h. With regard to SM, those with acyl chains of 18:0, 20:0, and 22:0 were lower in hypoxia and up to 1 h of reoxygenation, whereas all other SM species had higher rates in hypoxic and reoxygenated cells up to 1 h. At 12 h and 24 h, all SM ratios were similar between reoxygenated and normoxic cells. 

Overall, our data show that hypoxia and reoxygenation induce changes in the saturation levels of six lipid classes. Among them, TAG, DAG, PC, and CE were only affected after prolonged reoxygenation, while CER and SM were affected during and shortly after hypoxia. In addition, CER and SM classes not only exhibited differences between hypoxic and normoxic cells but also in their composition in specific lipid species, pointing to different enzymatic activities and/or signaling within SL metabolism.

## 4. Discussion

In this study, we set out to describe the metabolic rewiring of the CRC cell line HT29 when cultured in an oxygen-deprived environment and, more importantly, how this cell line responds to O_2_ restoration. HT29 has one of the most common oncogene mutations in the *PIK3CA* gene and has been found to exhibit resistance to hypoxia [34]. Here, we found that hypoxia increased glycolysis from an already-enhanced basal state [35]. The lack of oxygen also affected mitochondrial metabolism as we found almost all TCA cycle intermediates reduced. One exception to this was 2-OG, which was the most accumulated metabolite in hypoxic HT29. Beyond 2-OG, we also found a set of metabolites, namely GABA, 5-AVA, glutamate, aspartate, and proline, to be significantly reduced in hypoxia. Interestingly, restoring oxygen was not sufficient to completely normalize all hypoxia-induced metabolic changes. While our findings indicated a normoxia-like glycolysis and mitochondrial metabolism (e.g., TCA cycle activity) after 24 h of rexygenation, we also observed characteristic changes pointing to increased oxidation and ROS production and a surprising reduction of ATP levels. In contrast to the evident rewiring in central carbon metabolism, the lipidomics analysis resulted only in a small set of lipids being influenced by O_2_ deprivation, i.e., CER and HCER. However, our analysis revealed an underlying difference induced by hypoxic conditions, that is a significant change in the saturation level of the most abundant lipids, like TAG, DAG, PC, and CE.

It was recently found that the proliferation and survival of cancer cells carrying a *PIK3CA* mutation, as is HT29, is sensitive to the TCA cycle enzyme 2-oxoglutarate dehydrogenase (OGDH) [35]. This enzyme catalyzes the irreversible conversion of 2-OG and Coenzyme A-SH (CoA-SH) to succinyl-CoA and CO_2_ in an NAD^+^-dependent step. Suppression of OGDH has resulted in the accumulation of 2-OG, the reduction of the TCA cycle intermediates, succinate and fumarate as well as aspartate. The latter is connected to 2-OG via the malate-aspartate shuttle and transamination reactions occurring at both sides of the mitochondrial membrane, critical to maintain redox homeostasis and the regeneration of cytosolic NAD^+^. It has been shown in the same study [35] that excess of 2-OG in *PIK3CA* mutant cells deregulates the malate-aspartate shuttle, reduces the intracellular levels of aspartate and the NAD^+^/NADH ratio. In our work with HT29, we found hypoxia to induce an excess of 2-OG, significantly lower levels of aspartate, and a reduction of NAD^+^/NADH ratio up to 1 h of reoxygenation, compared to normoxic cells (Figure 2B). Thus, our data are consistent with a hypoxia-induced OGDH suppressed state. Interestingly, upon extended O_2_ restoration, 2-OG was reduced, its downstream TCA cycle metabolites succinate and fumarate as well as aspartate were increased, but the ratio of NAD^+^/NADH remained to the same levels as in hypoxia, RO-30m, and RO-1h (Figure S6). Regeneration of NAD^+^ occurs by ETC activity coupled to the synthesis of ATP, by the conversion of pyruvate to lactate, the malate-aspartate shuttle, and the desaturation of poly unsaturated fatty acids (PUFA) [36]. On the other hand, NAD^+^ can be consumed for glycolysis, in the TCA cycle, and in the oxidation of FAs. In our study, we observed that reoxygenated cells did not produce ATP at the same level as the normoxic cells, although the increased oxidation of glutathione suggests an increased ROS production via OXPHOS. However, after 24 h of reoxygenation, hypoxic HT29 produced more lactate compared to normoxic cells, suggesting a higher rate of conversion from pyruvate. Furthermore, reoxygenation induced significantly higher desaturation of TAG, DAG, PC, and CER compared to normoxic cells. Desaturation of FAs was recently described as a mechanism to regenerate cytosolic NAD^+^ when ETC or lactate production were impaired [37]. In our study, the latter condition does not apply for reoxygenated cells, while we observed the oxygen-dependent increase in desaturation. Thus, overall, we would expect reoxygenation to increase NAD^+^ regeneration, but this did not occur in our study (Figure S6). It is possible that the high glycolytic rates and the increase of TCA cycle activity consume NAD^+^, which counteracts its regeneration.

Together with aspartate, GABA was one of the most reduced metabolites in hypoxic HT29. GABA can be synthesized from glutamate like 2-OG or via the metabolism of polyamines, such as putrescine. The reaction of GABA with 2-OG results in the formation of succinic acid semialdehyde, which subsequently forms succinate, thus bypassing the formation of succinyl-CoA in the TCA cycle that requires one molecule of CoA. Although GABA has been extensively described as a neurotransmitter, it is known now that CRC cells including HT29 have GABA receptors, which upon activation via binding of GABA or its agonists, inhibit migration of cancer cells and induce their apoptosis [38,39]. In our study, GABA and both its possible precursors, glutamate and putrescine, were significantly lower in hypoxia but after reoxygenation gradually reached comparable levels to normoxic cells. It is noteworthy that 5-AVA, which was together with GABA aspartate and glutamate the most reduced metabolite in hypoxia, is also a weak GABA agonist via its binding to 4-aminobutyrate:2-oxoglutarate aminotransferase [36]. 5-AVA is the degradation product of lysine, and since *PIK3CA* mutant cells exhibit high expression of genes encoding lysine degradation [35], we can assume that 5-AVA synthesis is favored in normoxic condition. However, although normoxic HT29 maintained the high concentration of 5-AVA, the reoxygenated cells failed to reach this level even after 24 h of O_2_ restoration. In addition, we found lysine to be significantly higher in normoxic HT29 (Figure 1C), which cannot justify an increase in its degradation to produce 5-AVA. Nevertheless, to the best of our knowledge, this is the first time that 5-AVA is reported in hypoxic cancer cells to have such a significant difference compared to the normoxic culture. Taking together the GABA receptor apoptotic and antimigratory function, and the critical role of GABA and its agonists like 5-AVA, we hypothesize that GABA synthesis might be a possible target to further explore in the context of supporting cancer therapy.

As mentioned above, glutamate is the precursor of 2-OG in the mitochondria; the other source being the oxidation of pyruvate-derived citrate. A previous study has reported that glutaminolysis, and in particular the activity of glutamate pyruvate transaminase 2 (GPT2) that converts glutamate to 2-OG, is upregulated in *PIK3CA* mutants like HT29 and other CRC cell lines in hypoxia [40]. Thus, our findings of the significant reduction of glutamate together with the accumulation of 2-OG could be explained by overexpression of GPT2. Besides fueling TCA cycle via 2-OG, glutamate is also used for the synthesis of glutathione, together with glycine and cysteine. The precursor of the latter, cystine, was increasingly consumed by hypoxic cells, indicating an increased requirement for GSH as a cell defense to ROS. Indeed, we observed that upon prolonged reoxygenation, there was a decrease in GSH and an increase in GSSG, suggesting that O_2_ supply resulted in enhanced mitochondrial oxidative metabolism and the production of ROS. However, another use of cysteine (formed from extracellular cystine) is for the synthesis of hypotaurine, in an oxygen-dependent step [41]. Hypotaurine in turn is converted to taurine by the flavin-containing monooxygenase FMO 1, consuming either NADH or NADPH and oxygen. One of taurine’s functions is to act as an indirect antioxidant by enhancing the activities of enzymes which catalyze antioxidant scavenging. Another role of taurine is to counteract hypotaurine-induced proliferation and to induce apoptosis, as it was found upon extracellular taurine supplementation to HT29 [42]. Here, we found both hypotaurine and taurine to be increased in reoxygenated HT29, possibly due to increased O_2_ and NAD(P)H availability. Thus, based on taurine’s well-described anti-tumor progression properties when accumulated in CRC cells [42], targeting the hypotaurine–taurine reaction by increasing O_2_ availability might be a candidate to control tumor progression. 

In addition to the oxidation of glutathione, we also measured an increased production of betaine upon O_2_ restoration. Betaine is the product of choline oxidation at the expense of its phosphorylation and the synthesis of phosphocholine to support PC synthesis. Based on these findings, we can conclude that extended reoxygenation of hypoxic HT29 resulted in an increase in oxidation processes and possibly an increase in ROS production. The latter is not surprising, as it is one of the goals to support the effectiveness of chemotherapy and radiation therapy in cancer treatment.

Another important finding was the accumulation of intracellular creatine in reoxygenated cells even though it was equally consumed from the culture medium in all conditions of our experiment. Creatine metabolism involves its phosphorylation to phosphocreatine using one molecule of ATP. Phosphocreatine has a 50% higher energy for the γ-phosphate compared to ATP, and it provides an oxygen-independent pathway for energy. As such, it was recently found that phosphocreatine promotes highly hypoxic CRC tumor growth and metastasis and the creatine–phosphocreatine (Cr-PCr) metabolic axis is now being tested as a potential therapeutic target for CRC [43]. In agreement with this, we found that hypoxic HT29 had significantly higher phosphocreatine and lower ATP, thus utilizing the oxygen-independent phosphorylation of creatine to generate energy. When O_2_ availability increases, reoxygenated cells form phosphocreatine at comparable levels with the normoxic cells. In contrast, the already-lower ATP levels in hypoxic cells were further reduced in reoxygenation. ATP could be consumed for FA synthesis; however, we did not observe any increase in the total lipid pools. Another possibility is that hypoxic HT29 relies on lactate fermentation to produce ATP, and it retains this phenotype even when O_2_ is restored, in contrast to normoxic cells that can also produce ATP via ETC activity.

Our lipidomics analysis revealed underlying structural differences in lipids, as for example the increased desaturation of mainly TAG upon restoration of oxygen. While for most lipid classes, we did not identify notable differences in their total concentrations, CER exhibited significantly lower levels in hypoxic cells. Sphingolipid metabolism and ceramides in particular have gained attention recently in studies aiming to decipher the structural and signaling functions of lipids in CRC progression [33,44]. In general, increased CER is correlated to cell cycle arrest and apoptosis, while increased HCER and LCER contribute to tumor growth and metastasis. However, distinct species also exhibit different effects. For instance, increased concentration of CER(16:0) leads to apoptosis, in contrast to CER(24:0) and CER(24:1), which facilitate proliferation [44]. In our study, we found hypoxic HT29 to have lower levels of CER(16:0) and higher CER(24:0) and CER (24:1). This result might also contribute to the resistance of HT29 to hypoxic stress. Interestingly, upon restoration of O_2_, CER(16:0) increased to higher levels than normoxic HT29, thus pointing to a clear benefit of increased oxygen availability to halt the growth of this cell line. However, when we analyzed the product/substrate ratios between SLs with the same alkyl chain length, we saw a higher production of SM from CER in hypoxia and less HCER from CER. While the SM/CET ratio was similar between reoxygenated and normoxic cells after 24 h, the HCER species with longer FA chains, 22:0, 24:0, and 24:1, exhibited higher ratios at 24 h in RO compared to normoxic HT29. This might indicate a favorable adaptation of this cell line to abundant oxygen conditions; however, further functional studies are needed to support this finding.

This study was conducted on a single in vitro cultured cell line. As such, it is possible that the alterations we observed in central carbon and lipid metabolism could differ in an experimental animal model in vivo. Thus, further investigations are necessary to be performed in vivo to confirm the metabolic changes occurring in cancer cells from normoxia to hypoxia and vice versa during reoxygenation. These continuation studies could in turn facilitate the development of drugs targeting selected metabolic pathways aimed to improve the efficiency of anti-tumor therapies.

## Data Availability

All data used in this study are provided in Supplementary Materials.

## Supplementary Materials

The following supporting information can be downloaded at: https://www.mdpi.com/article/10.3390/metabo13070875/s1. Figure S1: Heatmap of standardized metabolite concentrations of HT29 in normoxia, hypoxia, RO-30m, and RO-1h. Figure S2: Heatmap of standardized concentrations of significant lipid species. Figure S3: Intracellular and extracellular concentrations of creatine, alanine, and creatine phosphate in hypoxic, normoxic, and reoxygenated HT29 up to 24 h. Figure S4: Saturation index (SI) for CE, FFA, LPC, LPE, and PE. Figure S5: Heatmap of standardized species/total class ratios for quantified sphingolipids. Figure S6: NAD^+^/NADH ratio as well the intracellular concentrations of NAD^+^ and NADPH in hypoxic, normoxic, and reoxygenated HT29 up to 24 h. Excel file with all data included in this study, intracellular metabolites, exchange of extracellular metabolites, total concentration of lipid classes, concentrations of all 513 lipid species, saturation and fold changes of TAG, and saturation indices for all lipid classes.
