# Supplementary material for: Metabolomic and Lipidomic Analysis of the Colorectal Adenocarcinoma Cell Line HT29 in Hypoxia and Reoxygenation
Source: Metabolites. 2023 Jul 23;13(7):875. doi: 10.3390/metabo13070875 (PMC10384744; doi:10.3390/metabo13070875)
Supplement: Supplementary file 1 [file metabolites-13-00875-s001.zip › Supplementary material.pdf]

Supplementary material

Figure S1. Heatmap of standardized metabolite concentrations of HT29 in normoxia, hypoxia, RO-30m and RO-1h. The subset of metabolites with $p<0.05$ (ANOVA, before multiple testing correction) are displayed. Star annotations indicate statistical significance after multiple testing correction, * $p<0.05$.

Figure S2. Heatmap of standardized concentrations of significant lipid species ($p<0.05$ before multiple testing correction). Only the subset of lipids with $p<0.05$ (ANOVA, before multiple testing correction) are displayed.

Figure S3. Intracellular and extracellular concentrations of creatine, alanine, and creatine phosphate in hypoxic, normoxic and reoxygenated HT29 up to 24h. Star annotations indicate statistical significance after multiple testing correction, * $p<0.05$, ** $p<0.01$, *** $p<0.001$.

Figure S4. Saturation Index (SI) for CE, FFA, LPC, LPE and PE; for SI definition see Methods. Star annotations indicate statistical significance after multiple testing correction, * $p<0.05$, ** $p<0.01$.

Figure S5. Heatmap of standardized species/total class ratios for quantified sphingolipids.

Figure S6. NAD^+/NADH ratio as well the intracellular concentrations of NAD^+ and NADPH in hypoxic, normoxic and reoxygenated HT29 up to 24h.
