# Supplementary figures and images for: Metabolomic and Lipidomic Analysis of the Colorectal Adenocarcinoma Cell Line HT29 in Hypoxia and Reoxygenation
Source: Metabolites. 2023 Jul 23;13(7):875. doi: 10.3390/metabo13070875 (PMC10384744; doi:10.3390/metabo13070875)

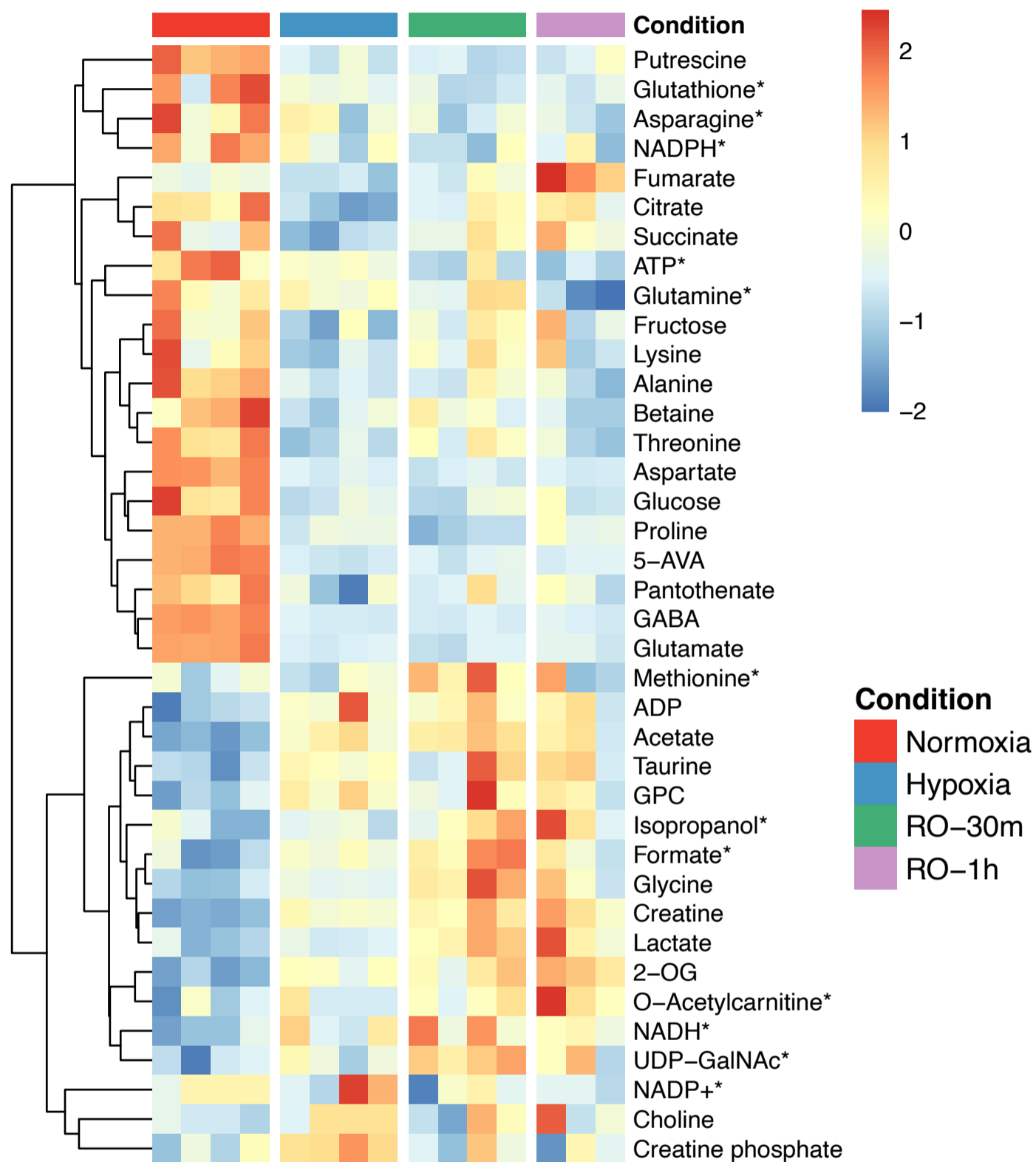

**Figure S1**

Supplement: Supplementary file 1 [file metabolites-13-00875-s001.zip › Figure S1.pdf]

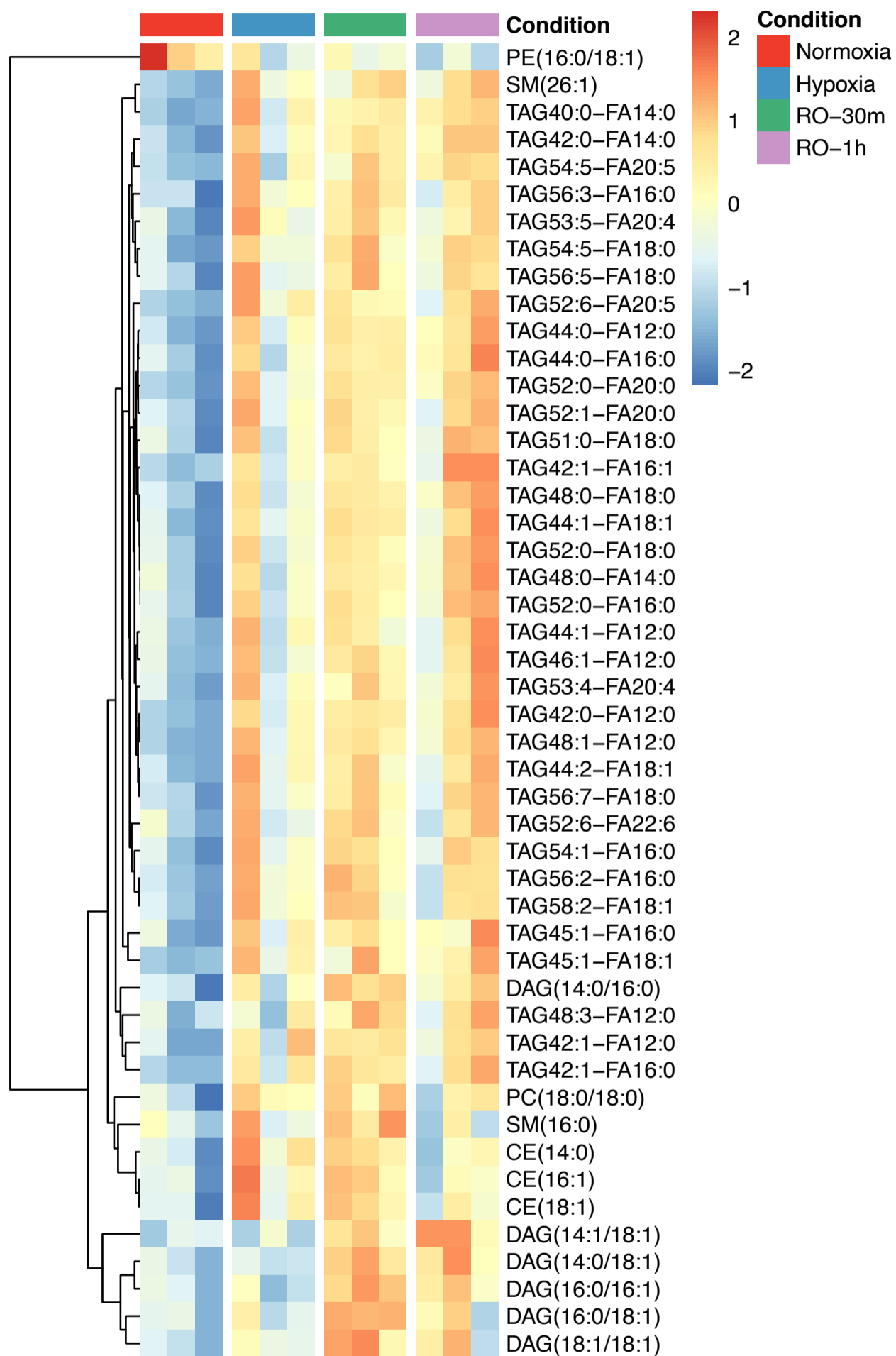

**Figure S2**

Supplement: Supplementary file 1 [file metabolites-13-00875-s001.zip › Figure S2.pdf]

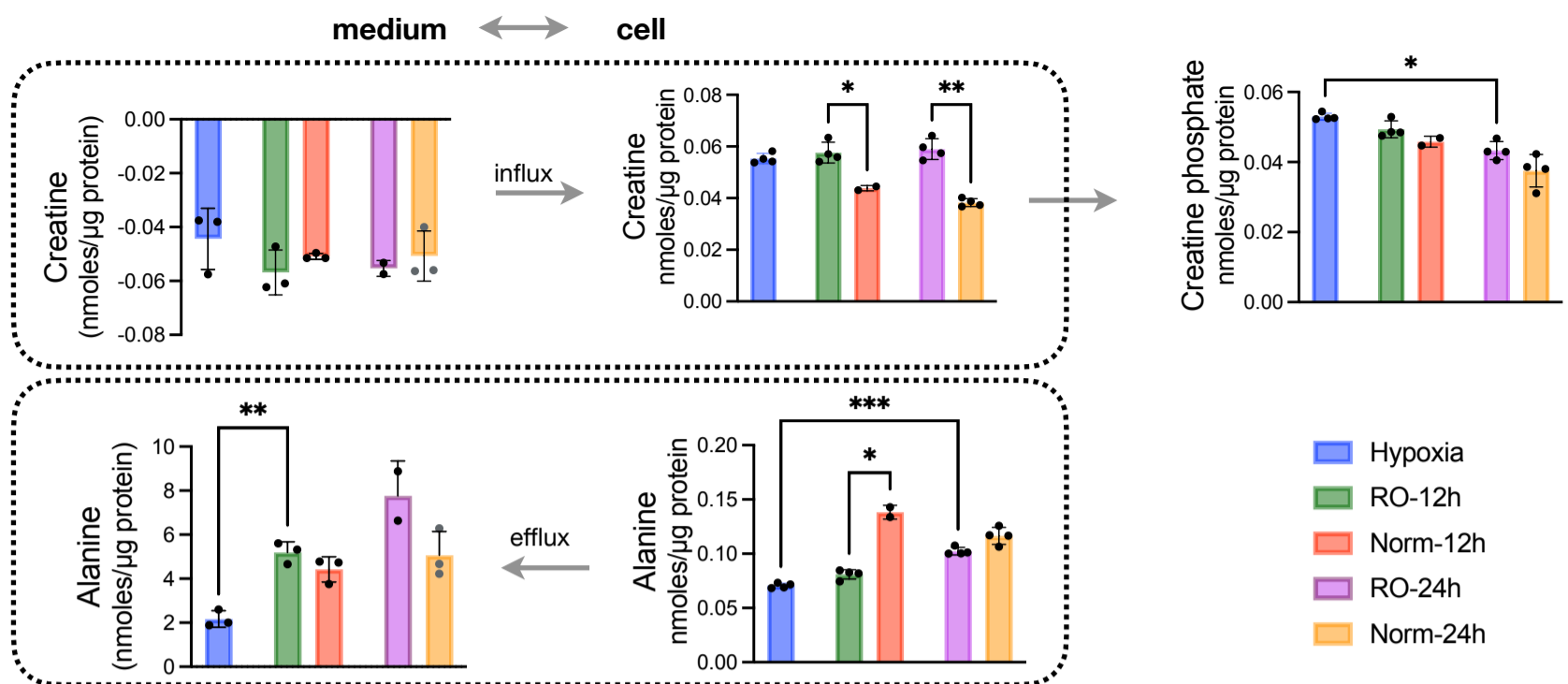

**Figure S3**

Supplement: Supplementary file 1 [file metabolites-13-00875-s001.zip › Figure S3.pdf]

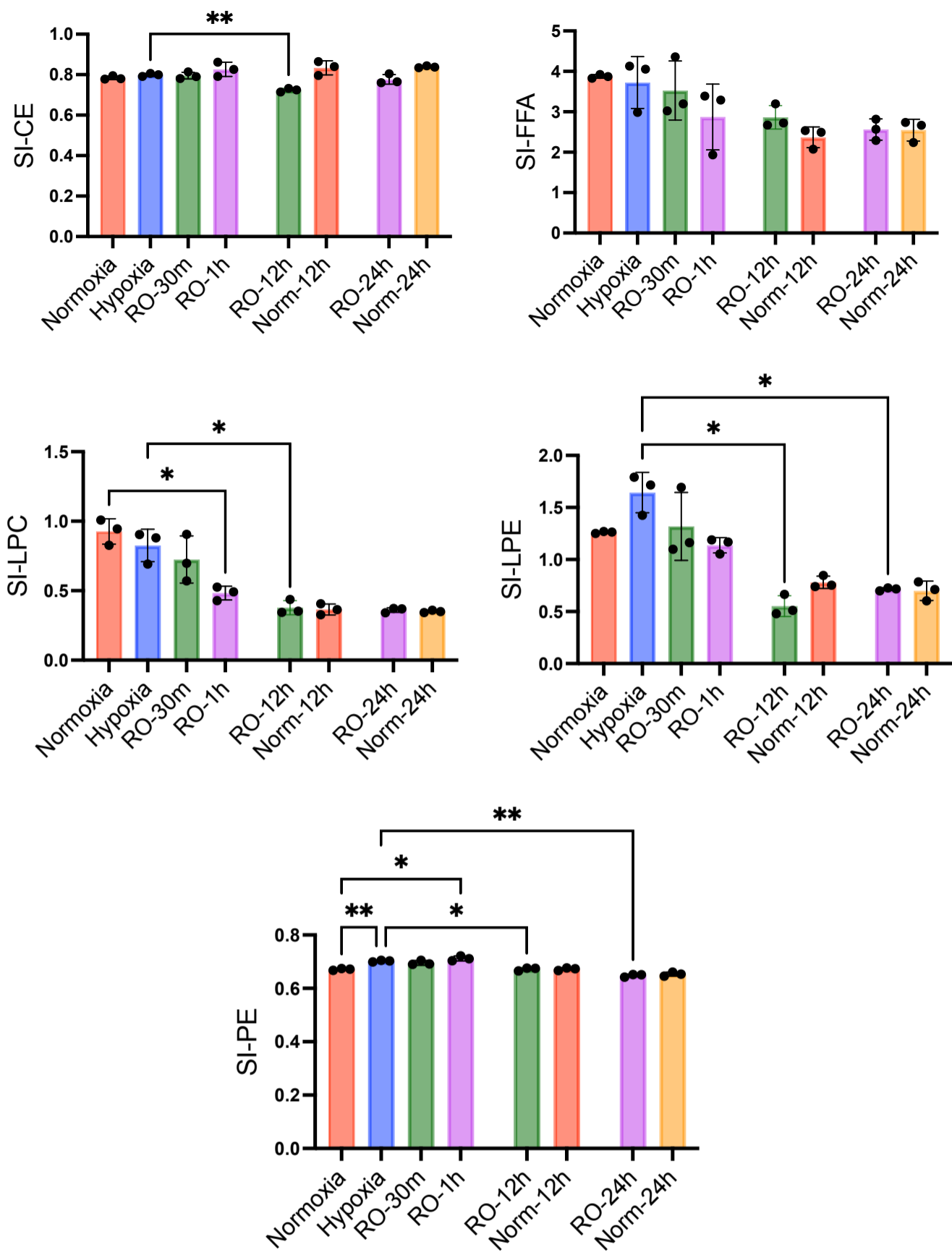

**Figure S4**

Supplement: Supplementary file 1 [file metabolites-13-00875-s001.zip › Figure S4.pdf]

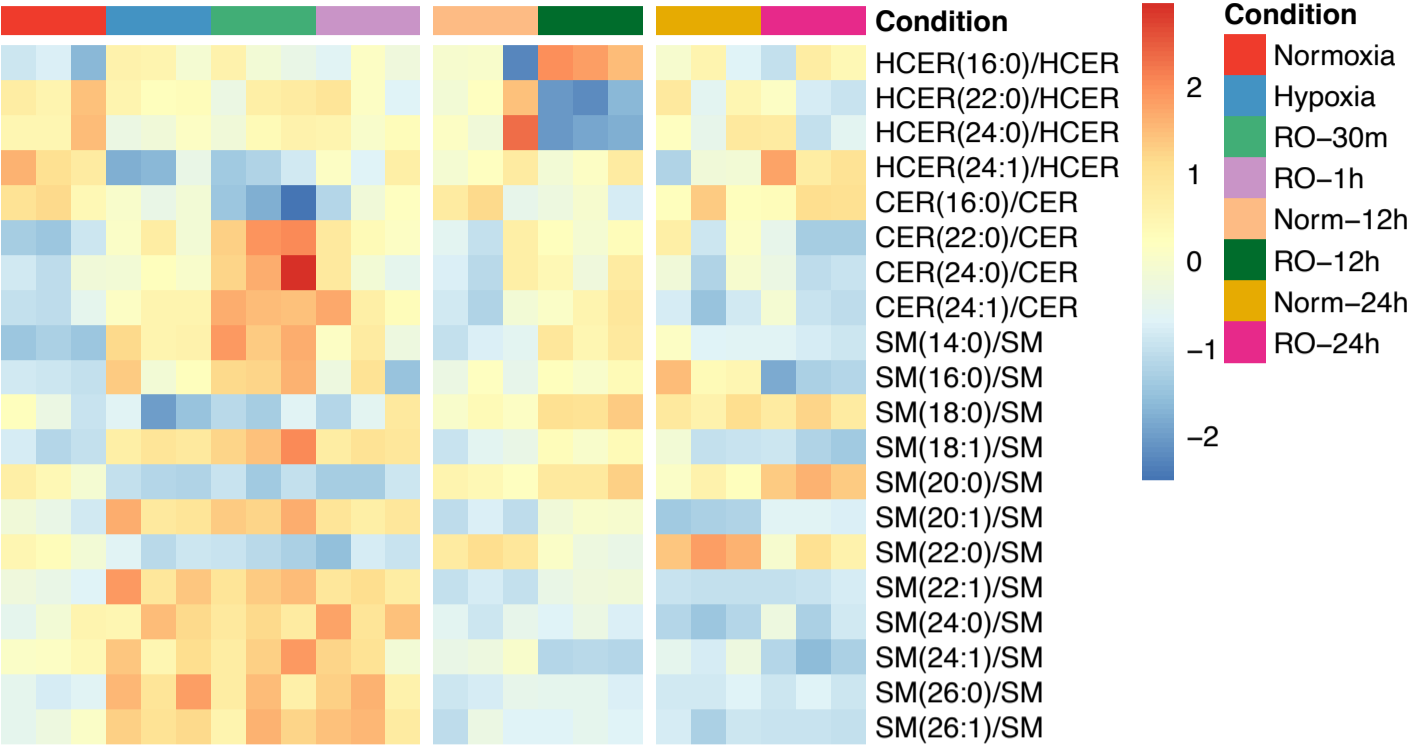

Figure S5

Supplement: Supplementary file 1 [file metabolites-13-00875-s001.zip › Figure S5.pdf]

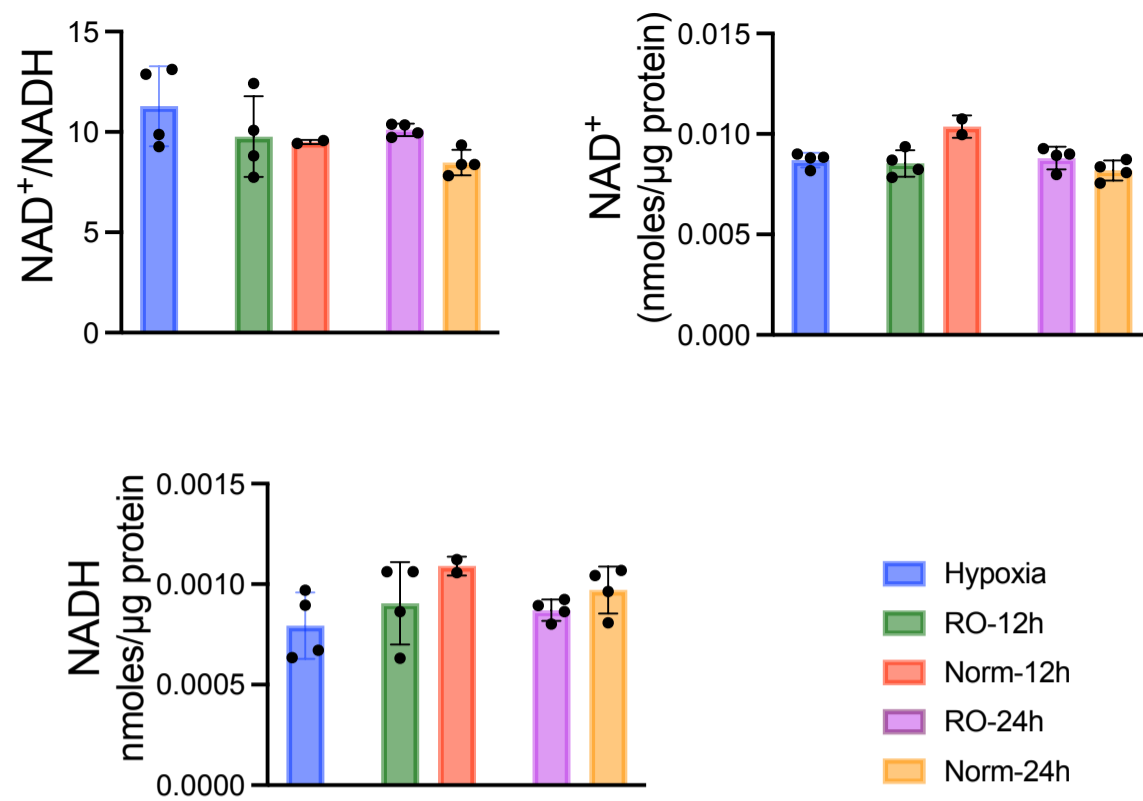

**Figure S6**

Supplement: Supplementary file 1 [file metabolites-13-00875-s001.zip › Figure S6.pdf]
